# A whole-genome assay identifies four principal gene functions that confer tolerance of meropenem stress upon *Escherichia coli*

**DOI:** 10.3389/frabi.2022.957942

**Published:** 2022-09-16

**Authors:** Nicholas M. Thomson, A. Keith Turner, Muhammad Yasir, Sarah Bastkowski, Martin Lott, Mark A. Webber, Ian G. Charles

**Affiliations:** ^1^ Microbes in the Food Chain, Quadram Institute Bioscience, Norwich, United Kingdom; ^2^ Norwich Medical School, University of East Anglia, Norwich, United Kingdom

**Keywords:** TraDIS-*Xpress*, *Escherichia coli*, meropenem, cell division, cell envelope, ATP, transcription, antibiotic tolerance

## Abstract

We report here the identification of four gene functions of principal importance for the tolerance of meropenem stress in *Escherichia coli*: cell division, cell envelope synthesis and maintenance, ATP metabolism, and transcription regulation. The primary mechanism of β-lactam antibiotics such as meropenem is inhibition of penicillin binding proteins, thus interfering with peptidoglycan crosslinking, weakening the cell envelope, and promoting cell lysis. However, recent systems biology approaches have revealed numerous downstream effects that are triggered by cell envelope damage and involve diverse cell processes. Subpopulations of persister cells can also arise, which can survive elevated concentrations of meropenem despite the absence of a specific resistance factor. We used Transposon-Directed Insertion Sequencing with inducible gene expression to simultaneously assay the effects of upregulation, downregulation, and disruption of every gene in a model *E. coli* strain on survival of exposure to four concentrations of meropenem. Automated Gene Functional Classification and manual categorization highlighted the importance at all meropenem concentrations of genes involved in peptidoglycan remodeling during cell division, suggesting that cell division is the primary function affected by meropenem. Genes involved in cell envelope synthesis and maintenance, ATP metabolism, and transcriptional regulation were generally important at higher meropenem concentrations, suggesting that these three functions are therefore secondary or downstream targets. Our analysis revealed the importance of multiple two-component signal transduction mechanisms, suggesting an as-yet unexplored coordinated transcriptional response to meropenem stress. The inclusion of an inducible, transposon-encoded promoter allowed sensitive detection of genes involved in proton transport, ATP production and tRNA synthesis, for which modulation of expression affects survival in the presence of meropenem: a finding that would not be possible with other technologies. We were also able to suggest new targets for future antibiotic development or for synergistic effects between gene or protein inhibitors and existing antibiotics. Overall, in a single massively parallel assay we were able to recapitulate many of the findings from decades of research into β-lactam antibiotics, add to the list of genes known to be important for meropenem tolerance, and categorize the four principal gene functions involved.

## Introduction

Meropenem is a bactericidal β-lactam antibiotic of the carbapenem class. Like other β-lactams, its primary mode of action is inhibition of penicillin binding proteins that mediate the crosslinking of peptidoglycan chains in the biogenesis of the cell wall (Drusano, 1997). This weakens the structure of the cell envelope and leads to cell lysis. In *Escherichia coli*, meropenem binds with greatest affinity to penicillin binding protein 2 (*mrdA*) and penicillin binding protein 4 (*dacB*), but also binds to PBP1a (*mrcA*), PBP1b (*mrcB*) and PBP3 (*ftsI*) (Davies et al., 2008; Kocaoglu and Carlson, 2015). Meropenem is highly resistant to degradation by many β-lactamases and cephalosporinases, has a broad spectrum of antibacterial activity and displays enhanced activity against Gram-negative bacteria compared to many other β-lactams (Yang et al., 1995). As such it is considered by the World Health Organization to be critical for human medicine.

Resistance to an antibiotic is the ability of a population of cells to grow at concentrations of antibiotic above a designated breakpoint minimum inhibitory concentration (MIC) (Balaban et al., 2019). However, cells also experience stress when exposed to concentrations below the MIC, and the study of their response to such conditions can provide insights into the biological mechanisms of antibiotic action and recalcitrance. Even above the MIC, some cells may display tolerance to antibiotics: surviving when other cells would not, despite the lack of a specific resistance factor; for example, due to slower growth (Dörr, 2021). Antibiotic tolerance can facilitate the evolution of resistance (Levin-Reisman et al., 2017; Windels et al., 2019) and is a contributary factor to the antibiotic resistance crisis (Church and McKillip, 2021). Subpopulations of tolerant cells that can survive treatment above the MIC of a bactericidal antibiotic and resume growth when exposure ends are known as persister cells (Balaban et al., 2019). An understanding of the separate but related mechanisms giving rise to antibiotic resistance, tolerance and persistence remains elusive, but a significant advance could be made by defining the sets of genes involved in each phenomenon: the “resistome”, “tolerome” and “persistome”. This would facilitate our ability to develop treatments to combat acute and recurrent bacterial infections (Fisher et al., 2017) and help to identify drug targets for use in combination therapies to potentiate the effects of existing antibiotics.

Transposon-Directed Insertion Sequencing with inducible gene expression (TraDIS-*Xpress*) provides a rapid and sensitive means to assess the contribution to survival in a defined growth condition of every gene within a genome by growing a pooled library of transposon-insertion mutants and quantifying the relative competitive success of every mutant (Langridge et al., 2009; Yasir et al., 2020). The mutant library used here was constructed by high frequency transposition of a mini-Tn5 derivative transposon containing a kanamycin resistance marker to allow selection for transformants, and an inducible promoter (*Ptac*). Typically, hundreds of thousands of mutants are generated and, because of the heterogeneity of Tn5 insertion site recognition, multiple transposon-insertion mutants will be created for every region of the genome. Transposon insertions within a gene will lead to inactivation of that gene by truncation or frame shift (termed “knockout insertions” here). Therefore, when the library is cultured, cells containing knockout insertions in genes that are important for growth in the selected conditions will exhibit differential growth and survival rates. Insertions at each location can be enumerated by sequencing, with changes in insertion frequency between conditions providing an indication of the relative importance of each genomic locus.

Additionally, when *Ptac* is induced, transposons located upstream of a gene will drive increased transcription, while transposons downstream of a gene will down-regulate transcription through RNA-interference effects. Therefore, TraDIS-*Xpress* can simultaneously assay the growth effects of inactivation, up-regulation, and down-regulation of every gene in the genome. We have found this approach to be powerful for assaying the roles of essential genes in stress responses based upon changes to expression (Yasir et al., 2020). Essential genes are often blind spots in traditional transposon mutagenesis studies as they cannot be inactivated.

Despite the long history of studying β-lactam antibiotics and their effects on cell physiology, we still have not arrived at a complete understanding of the mechanisms involved in killing and antagonism. In particular, the knock-on effects in multiple cell processes caused by acute damage to the peptidoglycan layer and the response to various concentrations of antibiotic have not been fully explored. We therefore applied TraDIS-*Xpress* to study the response of *E. coli* strain K-12 substrain BW25113 cells to a range of meropenem concentrations equivalent to 0.25×, 0.5×, 1× and 2× the MIC.

## Materials and methods

### Bacterial strains and growth conditions

The TraDIS-Xpress transposon mutant library was constructed in *E. coli* BW25113 and stored at −80°C in 15% glycerol; and has been described previously (Yasir et al., 2020). Briefly, a mini-Tn5 transposon encoding a kanamycin resistance cassette and an outward-transcribing, isopropyl-β-D-1-thiogalactopyranoside (IPTG)-inducible promoter (*Ptac*) was mixed with an EZ-Tn5 Transposase (Lucigen) and electroporated into competent cells. Transformants were selected on lysogeny agar plates containing kanamycin then collected and stored as a single pool. The library contains >700,000 unique transposon-insertion mutants.

Selection of transposon-insertion mutants in the presence of meropenem was performed in duplicate. Approximately 10^7^ c.f.u. from the library were grown overnight at 37°C in 1 mL of lysogeny broth containing meropenem at 2×, 1×, 0.5×, and 0.25× the MIC of 0.032 mg.mL^−1^. Control cultures without meropenem were grown in parallel. For each concentration of meropenem, cultures were grown in which 0 (uninduced), 0.2 or 1.0 mM IPTG was added.

### TraDIS-*Xpress* sequencing

DNA extraction and sequencing were carried out as described previously (Yasir et al., 2020). Briefly, after growing the cultures as described above, genomic DNA was extracted using a Quick-DNA Fungal/Bacterial 96 kit (Zymo Research) and fragmented using a Nextera tagmentation kit (Illumina). The fragmented DNA was amplified by PCR using oligonucleotide primers that hybridized at the transposon ends and contained an Illumina adapter sequence at their 5′ end. Sequencing of the resulting DNA libraries was done on a NextSeq 500 sequencing machine (Illumina) using a NextSeq 500/550 high output v2 kit for 75 cycles (Illumina).

### Insertion sequence data processing and analysis

Nucleotide read data were demultiplexed and converted to fastq format by bcl2fastq v. 2.20 (Illumina). Reads were then mapped to the *E. coli* BW25113 genome (CP009273) to identify the sites of transposon insertion using the Burrows-Wheeler Aligner (BWA) program (Li and Durbin, 2009) within Bio::TraDIS v. 1.4.1 (Barquist et al., 2016). The resulting transposon insertion site plots were manually inspected in the Artemis genome browser (Carver et al., 2012). AlbaTraDIS v. 0.0.5 (Page et al., 2020) was used to compare transposon insertion data between conditions. For each gene, insertions were counted within the coding sequence and within the 198 bp before and after the gene, with separate counts for the “forward”, “reverse” and “combined” orientations. This information was used to identify genes and adjacent regions with significantly changed transposon insertion patterns between condition and control. Data for the three IPTG induction concentrations were pooled and analyzed together for each meropenem concentration. This simplified downstream data analysis but allowed inspection of individual induction conditions if required.

AlbaTraDIS identified genes with significant changes in knockout insertions, insertions upstream of a gene (5′) and insertions downstream of a gene (3′) and provided values for the log_2_ fold change between control and condition (log_2_FC); number of reads located at each locus (counts) per million total reads for the sample, converted to log_2_ (log_2_CPM); a p-value of statistical significance; a q-value (the p-value adjusted for the false discovery rate); and a gene report suggesting the most likely interpretation of the data such as “knockout” (insertion within the coding region), “upregulated” (transcribed by *Ptac*) or “downregulated” (RNAi effect of *Ptac*). These data were further processed using Excel (Microsoft) and R v. 4.1.2 (R Core Team, 2021).

### Gene functional classification

Gene Functional Classification was performed using the Database for Annotation, Visualization and Integrated Discovery (DAVID) knowledgebase (Huang et al., 2009a; Huang et al., 2009b). The AlbaTraDIS output was filtered for knockout insertions for which at least one meropenem concentration met the criteria of q<0.0001 and log_2_CPM>3. This identified 296 gene names, which were uploaded to the DAVID website. The Gene Functional Classification tool was used to compare functional annotation terms for each gene in the list. A kappa distance score was calculated for every pair of genes, which governed the partitioning of genes into functional clusters using a fuzzy heuristic partitioning approach that allows genes to participate in more than one cluster to reflect the complexity of protein function. Kappa score calculation used a similarity term overlap = 4 and similarity threshold = 0.40. Classification into groups used an initial group membership = 5, final group membership = 5 and multiple linkage threshold = 0.60. *E. coli* strain K-12 substrain MG1655 was used as a reference genome to calculate an enrichment score for clustered genes.

### Relative antibiotic minimum inhibitory concentration and synergy measurement

These were performed using 96-well microplates with 200 µL lysogeny broth per well. Two-fold serial dilutions were used. Approximately 10^5^ c.f.u. of bacteria were added and incubated at 37°C overnight. The concentration of antibiotic at which the growth was inhibited >90% based on optical density was taken as the MIC. Antibacterial synergy between cefsulodin and meropenem was assessed similarly, employing combinations of concentrations of both and these were performed twice.

## Results

**Figure 1:**
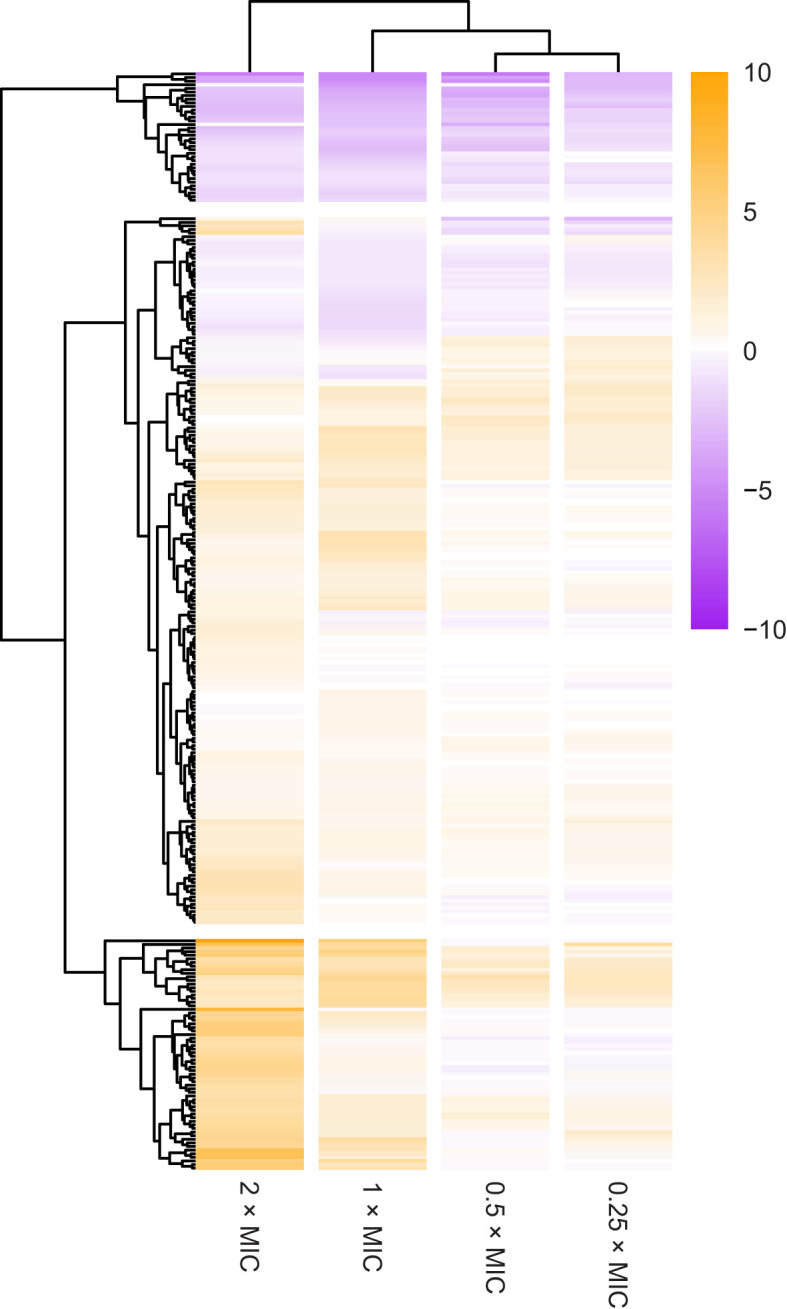
Overview of selection for transposon insertion mutants in the 296 most significant genes. The heatmap shows changes in number of transposon insertions identified within the coding sequence of each gene at each meropenem concentration tested, compared to control cultures without meropenem. Genes are ordered by hierarchical clustering by Euclidian distance of log_2_FC. Positive log_2_FC values indicate that transposon insertion mutants are selected for, suggesting that disruption of the gene is beneficial to survival under those conditions. Negative log_2_FC values indicate that transposon insertion mutants are selected against, suggesting that the gene is important for survival under those conditions.

More than 500 genes (q < 0.01) were identified as playing a role in the response of *E. coli* to meropenem based on phenotypes generated through knockout insertions, and upregulation or RNA interference from the transposon-encoded *Ptac* (**Supplementary Table 1**). However, to focus on the most significant hits, stricter cut-off parameters of q < 0.0001 and log_2_CPM > 3 for at least one meropenem concentration were used, ensuring all loci meeting these criteria had a highly significant change in transposon abundance between conditions and were represented by many mutants. This identified 296 genes with a very significant change in the number of knockout insertions. Hierarchical clustering by Euclidean distance of log_2_FC revealed robust clustering by meropenem concentration, suggesting an overall correlation between dose and effect of knockout mutations (**Figure 1**). Approximately two-thirds of the significant genes had a positive log_2_FC, indicating that knockouts of those genes tend to increase survival with meropenem. The other one-third had a negative log_2_FC in knockout insertions, indicating that loss of function of those genes lowers fitness for meropenem exposure.

Individual genes clustered into three main groups based on survival of knockout insertion mutants at different meropenem concentrations (**Figure 1**): 36 genes had negative log_2_FC values at all meropenem concentrations; 64 genes primarily had a positive log_2_FC and knockout insertions tended to increase tolerance more at 2× and 1×MIC than below the MIC; and the remaining 196 genes form a cluster containing the genes that had more moderate log_2_FC values, with a mixture of dose response patterns.

### Four principal gene functions are involved in meropenem tolerance

**Figure 2:**
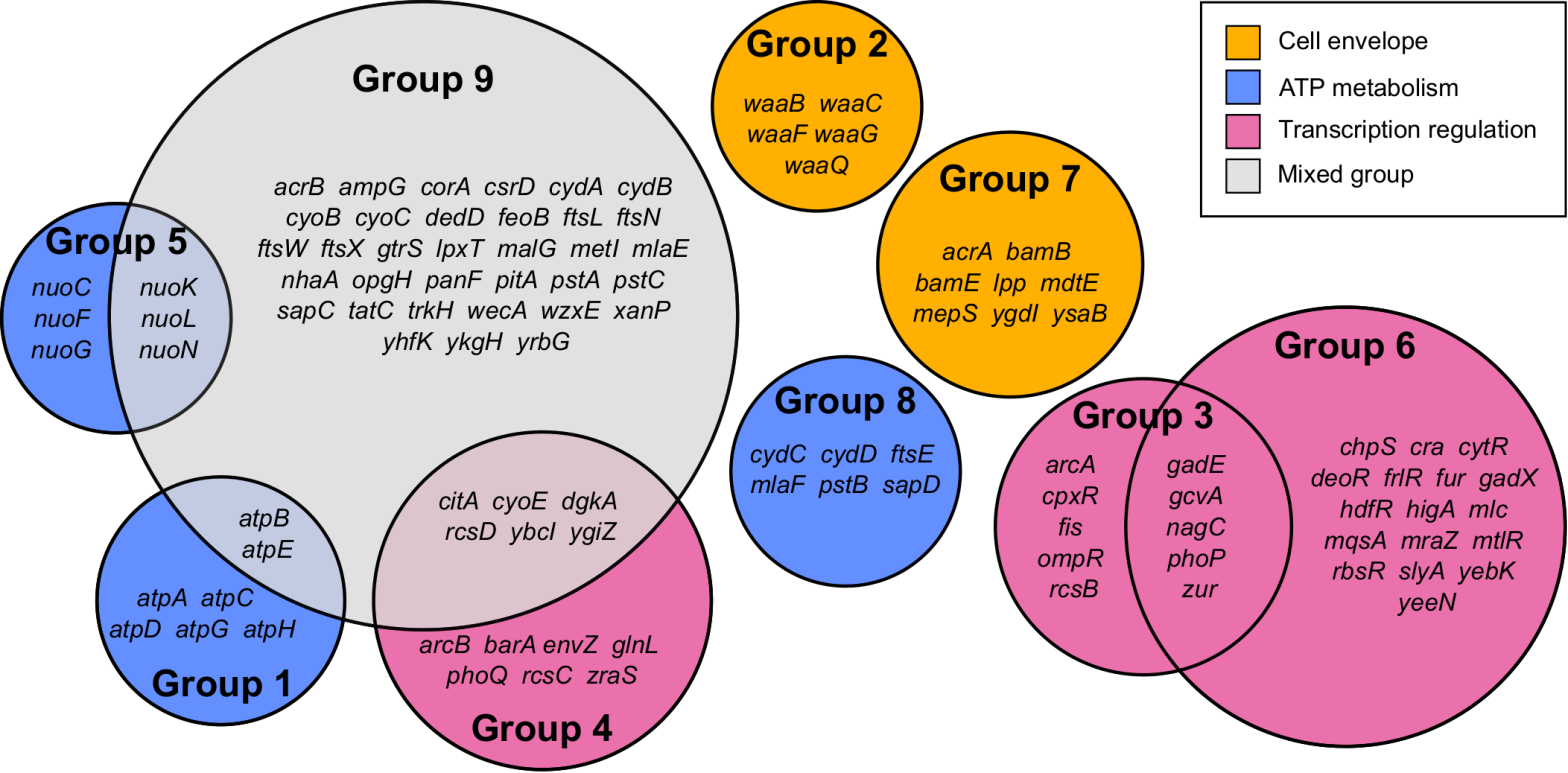
Summary of 108 genes comprising nine functional groups identified by Gene Functional Classification. The list of 296 most significant genes was compared to the *E. coli* str. K-12 substr. MG1655 genome to identify over-represented gene functions, which were then clustered by their categories in multiple databases.

**Figure 3:**
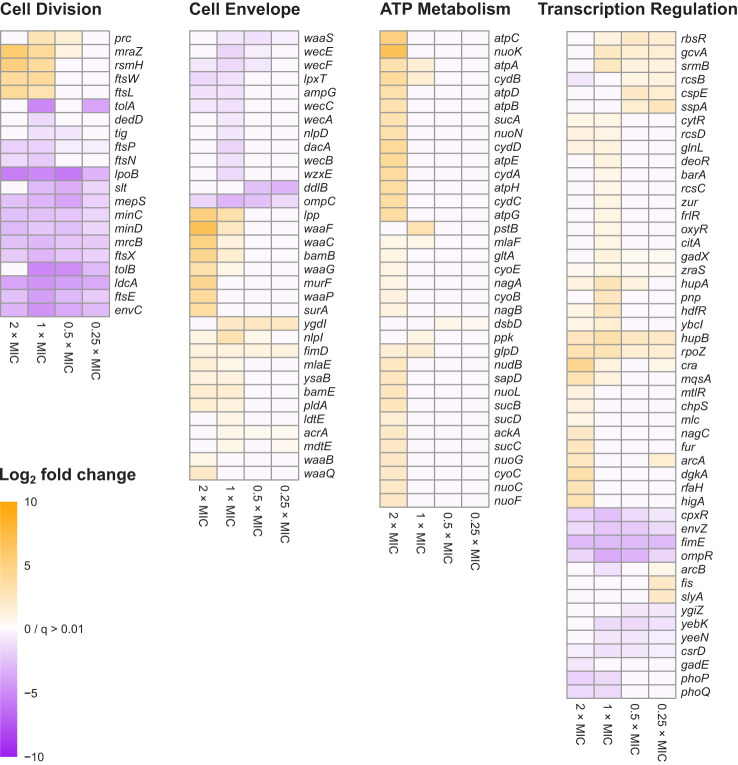
Heat map representations of log_2_FC for 138 genes belonging to the four principal gene functions involved in *E. coli* tolerance of meropenem stress. Principal gene functions were identified by Gene Functional Classification, as shown in **Figure 2**. Manual categorization of the 296 most significant genes (q<0.0001; log_2_CPM>3) then both expanded and refined the lists of genes belonging to each function. Cells are only colored when q<0.01 for the corresponding log_2_FC value. Genes in each heat map are arranged according to hierarchical clustering by Euclidian distance of log_2_FC, although for simplicity trees are not shown.

Gene Functional Classification using the 296 most significant genes classified 108 genes into nine functional groups whose functions were over-represented compared to the *E. coli* MG1655 genome (**Figure 2** and **Supplementary Table 2**). Eight of the nine groups could be assigned to one of three high-level functions that play the most significant roles in *E. coli* exposure to meropenem: cell envelope biogenesis, ATP metabolism, and regulation of transcription (**Figure 3**). Gene Functional Classification provides an overview of the most important groups of genes in an experiment. However, because it depends on annotation to cluster genes with similar functions it can overlook or falsely partition similar genes that have been annotated with different terms. For example, *nuo* and *atp* genes that all encode components of large enzyme complexes were placed in different groups based on their biochemical function within the enzyme complex (**Figure 2**).

Cell envelope biogenesis is represented by group 2, containing genes responsible for synthesis of the oligosaccharide core region of the lipopolysaccharide layer (*waaQGB* and *waaFC*, from two operons) and group 7, containing genes for lipoproteins with functions involved in the synthesis and structure of the outer membrane and peptidoglycan (murein) sacculus. ATP metabolism-related genes fell into three groups containing the *atpBEHAGDC* genes (group 1) for components of ATP synthase, the *nuoCFGKLN* genes (group 5) encoding components of respiratory complex I (NADH:ubiquinone oxidoreductase), and a cluster of 6 genes that all encode ATP-binding proteins (group 8). Meanwhile, three groups include genes that are involved in regulation of gene expression. Groups 3 and 4 may be considered as a pair because they predominantly include members of two-component regulatory systems, with one containing the transcriptional regulators *arcA, ompR, phoP* and *rcsB*, and the other containing their respective cognate signal transducers *arcB, envZ, phoQ* and *rcsCD*. Group 6 contains a broad range of transcription regulators; primarily transcriptional repressors, for which knockouts were beneficial to survival with meropenem, but also *gadE, phoP, yebK* and *yeeN*, for which knockouts were detrimental. The remaining cluster (group 9) contains 45 over-represented genes, mainly belonging to a mixture of the gene functions described above.

Manual inspection of the 187 unclustered genes identified other examples of genes belonging to these functional groups. Also apparent upon close inspection is that many of the most significant genes encode proteins that are involved in cell division. Gene Functional Classification did not identify a separate cluster for cell division because it categorizes genes according to the structural or enzymatic roles of their products, rather than the overall cell process. For example, *ftsE*, whose product powers the constriction of the mitotic septal ring (Arends et al., 2009), was clustered with other ATP-binding proteins, while the *ftsX* gene encoding the FtsE binding partner, is in the heterogeneous cluster of 45 over-represented genes together with *ftsL, ftsN* and *ftsW.* Genes for important peptidoglycan-modifying enzymes with key functions in cell division also fall into this category, including the cell division-specific penicillin binding protein 1b (*mrcB*) and *prc*, whose product cleaves the progenitor of penicillin binding protein 3 (FtsI) to form a mature protein that interacts with MrcB, FtsW and FtsZ. Therefore, cell division is a fourth important gene function in the response of *E. coli* to meropenem. **Figure 3** shows the log_2_FC for 138 genes belonging to the four principal gene functions following manual categorization of the 296 most significant genes.

### Cell division machinery is critical to meropenem tolerance

**Figure 4:**
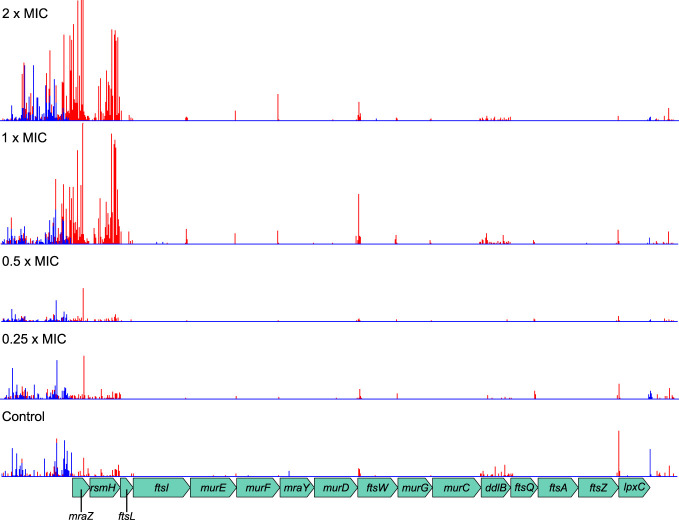
Transposon insertion pattern for the *dcw* gene cluster. Most genes in the cluster are essential and so transposon insertions within their coding sequences are not tolerated. However, insertions resulting in promotion of transcription from *Ptac* are beneficial to survival of meropenem treatment. Top to bottom: 2×MIC, 1×MIC, 0.5×MIC, 0.25×MIC, control. The red histogram indicates transposon insertions in which *Ptac* is oriented to transcribe from left to right, while the blue histogram indicates transposon insertions oriented to transcribe from right to left. For clarity, only one repeat of each concentration is displayed. Insertion patterns between repeats were highly similar.

The AlbaTraDIS workflow identified multiple members of the *dcw* gene cluster as important for growth during meropenem exposure. The *dcw* cluster is a transcriptional unit of 16 genes involved in cell division, which contains multiple transcriptional promoters but no terminator and thus may function as an operon (Vicente et al., 1998). The AlbaTraDIS results suggested that knockout insertions in some of these genes are beneficial under meropenem stress (**Figure 3**). However, inspection of the transposon insertion pattern within the *dcw* cluster revealed that the genes are essential (Goodall et al., 2018) and that transposon insertions were confined to specific regions towards the beginning and end of some genes and in intergenic spaces. Crucially, *Ptac* was always oriented in the same direction as the genes (**Figure 4**) showing that rather than knockout insertions, it is increased transcription of the *dcw* gene cluster which aids survival in the presence of meropenem. Furthermore, insertions in the same orientation before and within the dispensable genes *mraZ* and *rsmH* were highly beneficial because they are located at the start of the operon and therefore drive transcription of the whole operon.

The benefits of increased transcription were dependent on meropenem concentration, becoming particularly significant above the MIC. This is probably because the native levels of gene expression can cope with meropenem concentrations below the MIC, while increased levels are only required at or above the MIC. This result also illustrates the power of TraDIS-*Xpress* to reveal such detailed gene regulation effects as it is the only procedure able to assay transcription in this manner.

In contrast to the *dcw* cluster, knockout insertions in other genes central to cell division, such as *ftsE/X* and *minC/D* were detrimental to survival at all meropenem concentrations. Cell division is a highly dynamic process involving considerable re-modelling of the cell wall, and enzymes that are key to the recycling of peptidoglycan during this process, such as *ldcA* and *slt* were also important at all concentrations, as was *envC*, which encodes a factor that interacts with FtsEX and activates the peptidoglycan amidases AmiA and AmiB to enable septal splitting (Priyadarshini et al., 2007; Uehara et al., 2010; Cook et al., 2020). It is also notable that a larger proportion of the significant genes for cell division were important below the MIC of meropenem than for any of the other principal gene functions (**Figure 3**). Overall, this points to a critical role for the cell division machinery in the response to meropenem-induced stress.

### Dynamic remodeling of cell envelope is involved in meropenem tolerance

For cell envelope synthesis and remodeling genes that are not directly involved in cell division, there was an almost even split between genes for which knockout insertions reduced susceptibility to meropenem (positive log_2_FC), and those for which susceptibility was increased (negative log_2_FC). Overall, inactivation of these genes had a smaller effect than for cell division-related genes and tended to be important only at or above the MIC (**Figure 3**). This suggests that peptidoglycan remodeling proteins of the divisome may play a more important role in *E. coli* tolerance of meropenem than functionally similar proteins involved in other processes such as elongation.

Penicillin-binding proteins were not prominent within the data. Considering their central role in β-lactam tolerance, this is surprising. However, very small decreases in survival could be observed by manual inspection of knockout insertion sites for *mrcA* (PBP1a), *pbpC* (PBP1c) and *dacB* (PBP4), while *mrdA* (PBP2) and *ftsI* (PBP3) are essential genes and are therefore difficult to assay. *dacA* (PBP5) showed a significant negative log_2_FC at 1×MIC only. It is possible that the ability of meropenem to bind multiple PBPs means that inactivation of individual PBP genes have relatively small effects. The only PBP gene to show a significant log_2_FC at all meropenem concentrations was *mrcB* (PBP1b). As PBP1b provides transglycosylase and transpeptidase activity for the core enzyme complex of the divisome (Leclercq et al., 2017), this lends some support to the assertion that cell division-related genes have particular importance in meropenem tolerance.

**Figure 5:**
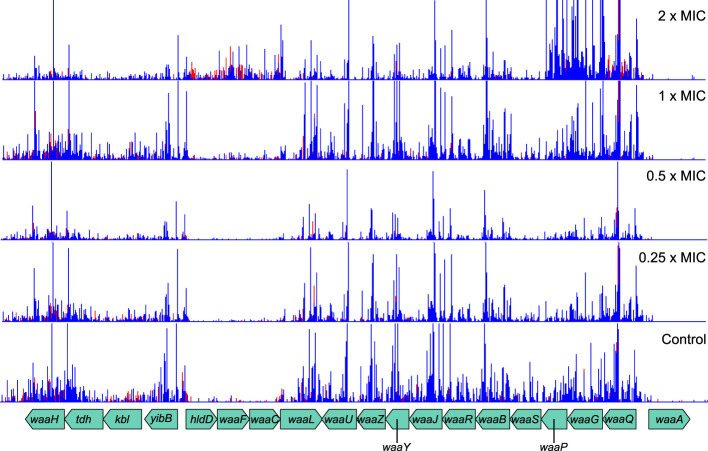
Transposon insertion site plots for the genomic region encoding the *waa* genes. While insertions in the essential *waaA* are not tolerated, insertions in other genes are selected for at high meropenem concentrations. Top to bottom: 2×MIC, 1×MIC, 0.5×MIC, 0.25×MIC, control. The red histogram indicates transposon insertions in which *Ptac* is oriented to transcribe from left to right, while the blue histogram indicates transposon insertions oriented to transcribe from right to left. For clarity, only one repeat of each concentration is displayed. Insertion patterns between repeats were highly similar.

In addition to peptidoglycan metabolism, the lipopolysaccharide layer—particularly the *waa* and *wec* gene clusters—was also important for meropenem tolerance (**Figure 3**). Products of *waa* genes catalyze the synthesis of the lipopolysaccharide core region, while *wec* gene products synthesize the enterobacterial common antigen, which attaches to the lipopolysaccharide (Rinno et al., 1980). The insertion site plots for the *waa* genes show that knockout insertions in *hldD/waaFC* in one operon, and in *waaQGP* in a second operon, are positively selected under high meropenem concentrations (**Figure 5**). Transposon insertions, many of which are expected to disrupt gene function, were tolerated throughout these two operons in our data, indicating that these genes are not essential (**Figure 5**). On the other hand, transposon insertions in *waaA* (in a third operon) were not detected because *waaA* is essential (Gerdes et al., 2003; Goodall et al., 2018) as it encodes 3-deoxy-D-manno-octulosonic acid transferase, which is fundamental to the production of lipid A (Belunis and Raetz, 1992; Klein et al., 2009). Therefore, it might be the case that interrupting certain genes leads to specific truncations of the lipopolysaccharide that facilitate survival at high meropenem concentrations (Joloba et al., 2004; Nakao et al., 2012), while complete loss of either the LPS or ECA is detrimental.

**Figure 6:**
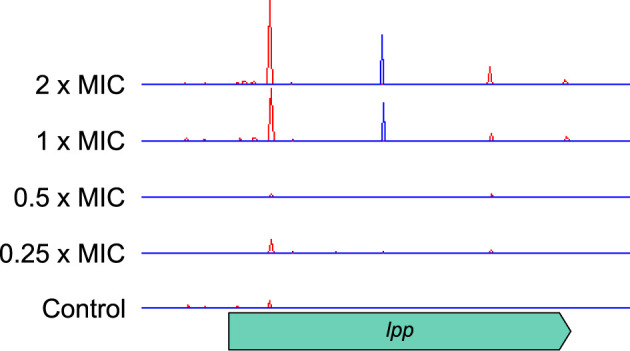
Transposon insertion site plots for the *lpp* gene and its flanking genes. Insertions are positively selected at high meropenem concentrations, with two locations, towards the start and centre of the gene, particularly favoured. Top to bottom: 2×MIC, 1×MIC, 0.5×MIC, 0.25×MIC, control. The red histogram indicates transposon insertions in which *Ptac* is oriented to transcribe from left to right, while the blue histogram indicates transposon insertions oriented to transcribe from right to left. For clarity, only one repeat of each concentration is displayed. Insertion patterns between repeats were highly similar.

One gene for which knockout insertions were surprisingly beneficial was *lpp*, which encodes a major peptidoglycan lipoprotein. Lpp forms a covalent link between the outer membrane and the peptidoglycan layer and has been estimated to account for up to 40% of peptidoglycan by mass (Braun and Rehn, 1969). Knockout insertions appeared to favor locations towards the start or center of the gene, suggesting that truncation rather than complete inactivation may be important for this effect (**Figure 6**). Loss of the cell wall and spheroplast formation is a strategy for β-lactam tolerance employed by *Pseudomonas aeruginosa* (Monahan et al., 2014) and Lpp regulates the mechanical properties of the cell envelope (Mathelié-Guinlet et al., 2020) so this observation might indicate a similar mechanism in *E. coli*.

The dose response of cell envelope genes was important, as they showed significant log_2_FC at the highest concentrations of meropenem (2×MIC and 1×MIC) but were not selected by exposures below the MIC. For example, *lpp* had log_2_FC values of 5.02 and 3.34 at 2×MIC and 1×MIC, respectively, but no significant change below the MIC. This might indicate a reduction in cell growth and division, and thus a reduced requirement for peptidoglycan and outer membrane synthesis at high meropenem concentrations. Alternatively, disruption of genes for preferred pathways might force cells to utilize alternative cell wall and membrane synthesis pathways that are less susceptible to meropenem.

### Maintenance of proton motive force aids survival with meropenem

The *atpBEHAGDC* and *nuoCFGKLN* genes identified by Gene Functional Classification operate on opposite sides of the ATP generation cycle. NADH:ubiquinone oxidoreductase, encoded by the *nuo* genes is the first complex in the electron transfer chain of respiration and uses the free energy derived from oxidation of NADH to expel protons from the cell. The F_0_F_1_ proton-translocating ATPase, encoded by the *atp* genes later harnesses the proton gradient thus accumulated to drive the production of ATP while allowing protons back into the cell. Although both complexes had significantly increased transposon insertions, again the ability to assay transcriptional effects on the genes is illuminating.

**Figure 7:**
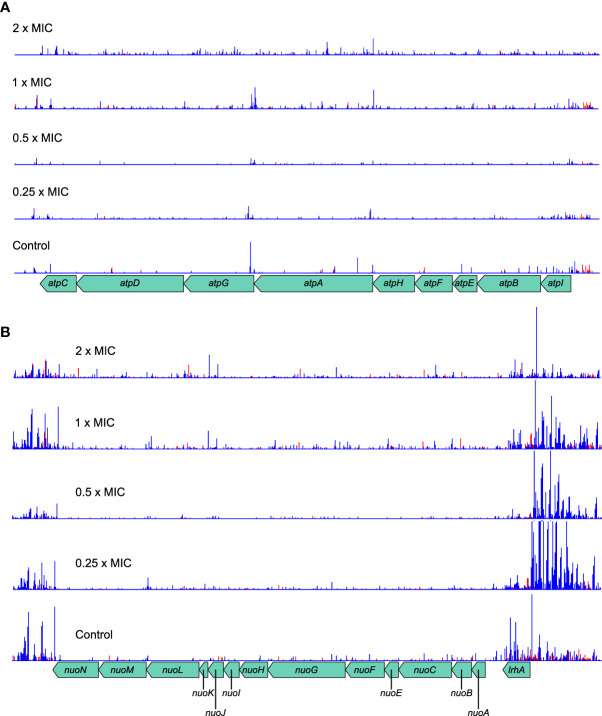
Transposon insertion patterns for the *atp* and *nuo* operons. **(A)** The *atp* operon tolerates transposon insertions in both orientations, which confer a selective advantage at high meropenem concentrations. Although the absolute number of insertions is low, the relative change at high concentrations is large. The overall effect is expected to decrease ATP synthesis activity of the F_0_F_1_ ATP synthase. **(B)** The *nuo* operon also tolerates insertions within the genes. However, insertions upstream of the operon, with *Ptac* oriented so as to transcribe the *nuo* genes, are highly favored in the presence of meropenem. The overall effect is expected to be increased activity of NADH:ubiquinone oxidoreductase, thus promoting the maintenance of the proton motive force. Top to bottom: 2×MIC, 1×MIC, 0.5×MIC, 0.25×MIC, control. The red histogram indicates transposon insertions in which *Ptac* is oriented to transcribe from left to right, while the blue histogram indicates transposon insertions oriented to transcribe from right to left. For clarity, only one repeat of each concentration is displayed. Insertion patterns between repeats were highly similar.

The *atp* operon tolerates insertions within genes in both directions and shows no effects of transcription from *Ptac* (**Figure 7A**). The log_2_FC is proportionally large, especially at higher meropenem concentrations, but this is partly because even the control sample has low absolute numbers of insertions. This probably arises as a balance is struck between maintaining the activity of ATP synthase for efficient energy generation and the beneficial effect of reducing that activity in the presence of meropenem.

The *nuo* operon shows a similar pattern of knockout insertions, with a slight preference for insertions at higher concentrations (**Figure 7B**). However, the region immediately upstream of the operon, containing *lrhA* and *alaA* is highly enriched in insertions oriented towards the *nuo* operon and therefore favoring its increased transcription. These insertions are particularly numerous at low concentrations, suggesting that NADH:ubiquinone oxidoreductase activity is more beneficial below the MIC for meropenem. Taken together, this suggests the greatest net benefit for meropenem tolerance is obtained by increasing *nuo* gene expression and minimizing *atp* gene expression. This seems most likely to have arisen as the result of a benefit gained through maintenance of the proton motive force as it would maintain proton export but minimize their import, thereby shifting the balance of metabolic flux away from ATP metabolism.

This hypothesis is supported by the identification of Gene Functional Classification group 3 containing ATP-binding proteins, for which knockout insertions confer increased tolerance of meropenem (**Figure 2**). Cells that minimize non-essential energy consumption through these proteins might reduce demand for ATP, thus preserving the proton motive force. This may also facilitate slowing cell growth to reduce the dependence on peptidoglycan synthesis and remodeling, and prevent extra stress being imposed upon the cell by reactive oxygen species that are generated during oxidative phosphorylation.

### Global changes in gene regulation

AlbaTraDIS identified a wide range of transcriptional regulators and related genes, which exhibited varying transposon insertion patterns. As identified by Gene Functional Classification, an important class of transcription-regulating genes were those belonging to two-component signal transduction systems (**Figure 2** and **Supplementary Table 2**). Four transducer-regulator pairs were identified, while the transcriptional regulator *cpxR* and the signal transducers *barA, citA, glnL* and *zraS* were identified without their partners. Interestingly, knockout insertions in the *arcA/B* and *rcsB/CD* genes were beneficial to survival with meropenem (positive log_2_FC) while knockout insertions in the *ompR/envZ* and *phoP/Q* systems were detrimental (negative log_2_FC). The *ompR/envZ* and *cpxR* genes had significant log_2_FC values at all concentrations, suggesting that they might play more fundamental roles in meropenem tolerance than the other systems. Both systems are involved in the response to cell envelope damage.

Global transcriptional regulators were also identified, including *fis* [regulation of up to 21% of genes (Cho et al., 2008)], *zur* (Zn-binding transcriptional repressor) and *cra* (repressor-activator for carbon metabolism). However, the effects of these knockout insertions were only significant at specific meropenem concentrations. Therefore, large-scale transcriptional regulation of multiple systems may provide some benefit by, for example, reducing cell growth rate, but is unlikely to be a key factor in meropenem tolerance. Instead, more targeted responses such as those mediated by the two-component systems identified above are more likely to be important.

### Influence of the stringent response on meropenem tolerance

Separate from transcriptional control of gene expression, the *E. coli* stringent response increases survival under a range of starvation conditions. Increased intracellular concentrations of (p)ppGpp inhibit rRNA and tRNA production, thus attenuating the rates of transcription, translation, and cell growth (Srivatsan and Wang, 2008). The key genes involved in regulating (p)ppGpp levels are *relA*, whose product produces ppGpp from GDP and ATP, and *spoT*, whose product can produce (p)ppGpp but also recycles it back to GTP or GDP. In this experiment, *spoT* knockout mutants showed increased tolerance to high concentrations of meropenem, suggesting a role for the stringent response in antibiotic tolerance. *relA* knockout insertions had no significant effect; however, 5′ transcription-enhancing insertions in the upstream *rlmD* gene also increased tolerance to high concentrations of meropenem. Therefore, increased levels of *relA* and decreased levels of *spoT* are beneficial to survival with meropenem. Furthermore, knockout insertions in *rpoS*, which mediates the general stress response and is enhanced by high (p)ppGpp levels (Lelong et al., 2007; Battesti et al., 2011), were also detrimental at high meropenem concentrations.

### Transcriptional interference of tRNA genes

**Figure 8:**
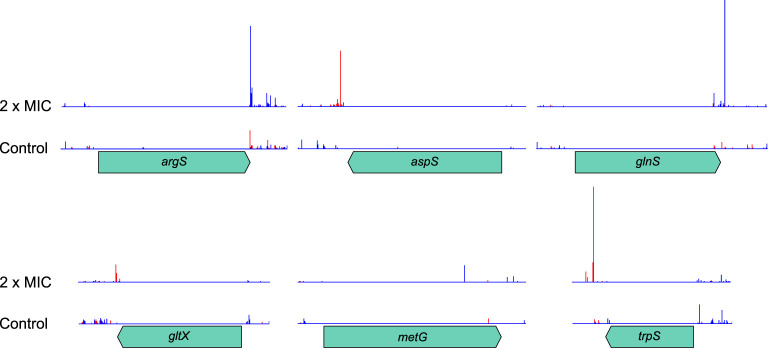
Transposon insertion site plots for the six tRNA synthetase genes with significant transcriptional interference. Insertions are not tolerated within the coding region of any of the genes. However, downstream insertions oriented such that transcription from *Ptac* proceeds in the opposite direction to the synthetase gene are selected for at high meropenem concentrations. The red histogram indicates transposon insertions in which *Ptac* is oriented to transcribe from left to right, while the blue histogram indicates transposon insertions oriented to transcribe from right to left. For clarity, histograms are only shown for the control and 2 × MIC samples.

In an expedient demonstration of the unique capabilities of TraDIS-*Xpress* both to assay essential genes and to determine transcriptional effects, we identified that RNA interference of six aminoacyl-tRNA synthetase genes was beneficial to survival at high concentrations of meropenem (**Table 1**). Although knockout insertions were not tolerated, transposon insertions downstream of the genes and oriented such that *Ptac* transcribed the gene in reverse were selected for (**Figure 8**). This indicates that RNA interference caused by transcription from the *lac* promoter decreased expression of these genes and led to a fitness benefit. The effect of such insertions was large (1.9 – 11.0 log_2_FC), but only at or above the MIC. Interestingly, the stringent response acts partly through suppressing tRNA synthesis, so decreasing tRNA-related gene transcription in this way might have similar effects in a non-(p)ppGpp-dependent manner. Disruption of the C-terminus of MetG by insertion of a transposon increases persister cell formation by 10,000× compared to the wild-type (Girgis et al., 2012).

**Table 1:** tRNA synthetase genes showing transcriptional interference from the mini Tn5-encoded *Ptac*.

Gene	Protein	Log_2_FC at 2/1/0.5/0.25 MIC
*argS*	arginyl-tRNA synthetase	8.0/4.5/-/-
*aspS*	aspartyl-tRNA synthetase	8.6/0/-/-
*glnS*	glutamyl-tRNA synthetase	7.1/3.7/-/-
*gltX*	glutamyl tRNA synthetase	2.5/-/-/-
*metG*	methionyl-tRNA synthetase	11.0/-/-/-
*trpS*	tryptophanyl-tRNA synthetase	3.9/1.9/-/-

Non-significant log_2_FC values (q>0.0001 and/or log_2_CPM<3) are indicated by a dash.

### Identification of potential gene targets for meropenem-synergizing drugs

Identifying the full range of genes contributing to meropenem susceptibility allows a rational approach to predicting antibiotic combinations which act additively or synergistically. Genes for which knockout insertions cause the most significant decreases in log_2_FC should be the best targets for drugs that will provide a synergistic effect when administered in combination with meropenem (**Table 2**). The gene with the largest such effect was *lpoB*, encoding an outer membrane lipoprotein that binds to and stimulates penicillin binding protein 1b (*mrcB;* also identified by this experiment as a potential drug target). The next largest effects were seen for *tolA* and *tolB*, both of which encode members of the Tol-Pal system, with roles in outer membrane invagination and peptidoglycan processing during cell division. However, the effects were not significant at all meropenem concentrations, raising doubts about their suitability as drug targets. Knockout insertions in genes for other peptidoglycan-processing proteins such as *ldcA, envC, mepS* and *slt* did show significantly increased susceptibility at all concentrations and so are more promising targets (together with the amidases *amiA* and *amiB* that EnvC activates, although these genes were not identified as significant in this experiment).

**Table 2:** Genes with the largest log_2_FC in knockout insertions (positive and negative presented separately) selected by meropenem treatment.

Locus	Log_2_FC at 2/1/0.5/0.25 MIC	Operon	Annotation
*Top 25 genes for which knockout insertions were beneficial (positive log2FC), ranked by log_2_FC*			
*rpsA*	11.2/-/-/-	>+rpsA	30S ribosomal subunit protein S1. Structural component; Ribosomal proteins - synthesis, modification.
*lipA*	8/-/-/-	-lipA<	Lipoate synthase. Putative enzyme; Biosynthesis of cofactors, Carriers: Lipoate.
*cysE*	6.7/-/-/-	-cysE -gpsA -secB -grxC<	Serine acetyltransferase. Enzyme; Amino acid biosynthesis: Cysteine.
*waaF*	6.6/2.4/-/-	>+hldD +waaF +waaC +waaL	ADP-heptose:LPS heptosyltransferase II. Enzyme; Macromolecule metabolism: Lipopolysaccharide.
*nuoK*	6.6/-/-/-	-nuoL -nuoK -nuoJ -nuoI -nuoH -nuoG -nuoF -nuoE -nuoC<	NADH:ubiquinone oxidoreductase, membrane subunit K. Enzyme; Energy metabolism, carbon: Aerobic respiration.
*gmhB*	6.6/-/-/-	>+gmhB	D,D-heptose 1,7-bisphosphate phosphatase. Enzyme; Surface polysaccharides and antigens.
*mtn*	5.7/-/-/-	-yadS -btuF -mtn<	5’-methylthioadenosine/S-adenosylhomocysteine nucleosidase.
*mraZ*	5.5/4.2/-/-	>+mraZ +rsmH +ftsL +ftsI +murE +murF +mraY +murD +ftsW +murG +murC +ddlB +ftsQ +ftsA +ftsZ +lpxC	RsmH methytransferase inhibitor.
*glnA*	5.4/4.8/-/-	-glnA<	Glutamine synthetase. Enzyme; Amino acid biosynthesis: Glutamine.
*ispB*	5.4/-/-/-	>+ispB	Octaprenyl diphosphate synthase. Enzyme; Biosynthesis of cofactors, carriers: Menaquinone, ubiquinone.
*ratA*	5.3/-/-/-	-ratB -ratA<	Toxic UPF0083 family protein inhibitor of 70S ribosome formation.
*spoT*	5.3/1.6/-/-	>+gmk +rpoZ +spoT +trmH +recG	Bifunctional (p)ppGpp synthetase II/guanosine-3’,5’-bis pyrophosphate 3’-pyrophosphohydrolase. Enzyme; Global regulatory functions.
*atpC*	5.2/-/-/-	-atpC -atpD -atpG -atpA -atpH -atpF -atpE -atpB -atpI<	F1 sector of membrane-bound ATP synthase, epsilon subunit. Enzyme; ATP-proton motive force interconversion.
*rsmH*	5.2/3.9/-/-	>+mraZ +rsmH +ftsL +ftsI +murE +murF +mraY +murD +ftsW +murG +murC +ddlB +ftsQ +ftsA +ftsZ +lpxC	16S rRNA m(4)C1402 methyltransferase, sAM-dependent. Enzyme; rRNA modification.
*ubiF*	5.1/-/-/-	>+ubiF	2-octaprenyl-3-methyl-6-methoxy-1,4-benzoquinol oxygenase.
*chpB*	5.1/1.6/-/-	>+chpS +chpB	Toxin of the ChpB-ChpS toxin-antitoxin system. Putative factor; Not classified.
*lpp*	5/3.3/-/-	>+lpp	Murein lipoprotein. Membrane; Murein sacculus, peptidoglycan.
*waaC*	4.9/-/-/-	>+hldD +waaF +waaC +waaL	ADP-heptose:LPS heptosyl transferase I. Enzyme; Macromolecule metabolism: Lipopolysaccharide.
*mnmA*	-/4.8/-/-	-purB -hflD -mnmA -nudJ -rluE<	tRNA(Gln,Lys,Glu) U34 2-thiouridylase, first step in mnm(5)-s(2)U34-tRNA synthesis. Enzyme; tRNA modification.
*glnV*	4.6/-/-/-	-glnX -glnV -metU -glnW -glnU -leuW -metT<	tRNA-Gln.
*galU*	4.6/-/-/-	>+galU	Glucose-1-phosphate uridylyltransferase. enzyme; Degradation of small molecules: Carbon compounds.
*purA*	4.6/-/-/-	>+purA	Adenylosuccinate synthetase. Enzyme; Purine ribonucleotide biosynthesis.
*hldD*	4.5/-/-/-	>+hldD +waaF +waaC +waaL	ADP-L-glycero-D-mannoheptose-6-epimerase, NAD(P)-binding. Enzyme; Surface polysaccharides and antigens.
*rnc*	4.4/3.8/-/-	>+arnB +arnC +arnA +arnD +arnT +arnE	RNase III. Enzyme; Degradation of RNA.
murF	4.3/-/-/-	>+mraZ +rsmH +ftsL +ftsI +murE +murF +mraY +murD +ftsW +murG +murC +ddlB +ftsQ +ftsA +ftsZ +lpxC	UDP-N-acetylmuramoyl-tripeptide:D-alanyl-D-alanine ligase. Enzyme; Murein sacculus, peptidoglycan.
*Top 25 genes for which knockout insertions were detrimental (negative log2FC), ranked by log_2_FC*			
*lpoB*	-5.5/-5.2/-6/-	>+hinT +ycfL +lpoB +thiK +nagZ +ycfP	OM lipoprotein stimulator of MrcB transpeptidase. Murein sacculus, peptidoglycan.
*tolB*	-/-5.2/-4.9/-	>+tolB +pal +ybgF	Periplasmic protein. Factor; Colicin-related functions.
*tolA*	-/-5/-/-	>+ybgC +tolQ +tolR +tolA	Membrane anchored protein in TolA-TolQ-TolR complex. Membrane; Colicin-related functions.
*ldcA*	-3.9/-4.7/-4.2/-3.5	-cvrA -ldcA<	Murein tetrapeptide carboxypeptidase; LD-carboxypeptidase A. Enzyme; Murein sacculus, peptidoglycan.
*envC*	-3.1/-4.5/-3.5/-2.8	>+gpmM +envC	Activator of AmiB,C murein hydrolases, septal ring factor. Enzyme; Murein sacculus, peptidoglycan.
*ftsE*	-2.6/-4.1/-3/-2.1	-ftsX -ftsE -ftsY<	Putative ABC superfamily transporter ATP-binding subunit. Phenotype; Not classified.
*ispD*	-2.3/-3.9/-3.4/-3.2	-pcm -umpG -truD -ispF -ispD -ftsB<	4-diphosphocytidyl-2C-methyl-D-erythritol synthase.
*mepS*	-2.7/-3/-3.9/-	>+mepS	Murein DD-endopeptidase, space-maker hydrolase, mutational suppressor of prc thermosensitivity, outer membrane lipoprotein. Enzyme; Murein sacculus, peptidoglycan.
*ompR*	-1.7/-3.6/-3/-1.7	-envZ -ompR<	Response regulator in two-component regulatory system with EnvZ. Regulator; Global regulatory functions.
*slt*	-/-2.9/-3.4/-1.6	>+slt +trpR	Lytic murein transglycosylase, soluble. Enzyme; Murein sacculus, peptidoglycan.
*ftsX*	-2.2/-3.2/-2.4/-1.8	-ftsX -ftsE -ftsY<	Inner membrane putative ABC superfamily transporter permease. Membrane; Cell division.
*ddlB*	-/-/-/-3.1	>+mraZ +rsmH +ftsL +ftsI +murE +murF +mraY +murD +ftsW +murG +murC +ddlB +ftsQ +ftsA +ftsZ +lpxC	D-alanine:D-alanine ligase. Enzyme; Murein sacculus, peptidoglycan.
*ompC*	-1.7/-3/-2.5/-1.4	-ompC<	Outer membrane porin protein C. Membrane; Outer membrane constituents.
*fimE*	-3/-2.8/-2.8/-3	>+fimE	Tyrosine recombinase/inversion of on/off regulator of fimA. Regulator; Surface structures.
*mrcB*	-2.7/-2.9/-2.3/-2.1	>+mrcB	Fused glycosyl transferase and transpeptidase. Enzyme; Murein sacculus, peptidoglycan synthetase; penicillin-binding protein 1B.
*bhsA*	-2.9/-/-/-	>+bhsA	Biofilm, cell surface and signaling protein.
*minD*	-2.8/-2.4/-2.5/-1.5	-minE -minD -minC<	Membrane ATPase of the MinC-MinD-MinE system. Enzyme; Cell division.
*cpxR*	-1.8/-2.7/-1.3/–.9	-cpxA -cpxR<	Response regulator in two-component regulatory system with CpxA. Putative regulator; Not classified.
*minC*	-2.6/-2.7/-2.4/-1.6	-minE -minD -minC<	Cell division inhibitor. Factor; Cell division
*fabF*	-/-2.7/-/-	>+acpP +fabF	3-oxoacyl-[acyl-carrier-protein] synthase II. Enzyme; Fatty acid and phosphatidic acid biosynthesis.
*tatC*	-1.3/-2.6/-2.4/-1.9	>+rmuC +ubiE +ubiJ +esrE +ubiB +tatA +tatB +tatC	TatABCE protein translocation system subunit.
*yibN*	-1.7/-2.6/-2.6/-	-yibN<	Putative rhodanese-related sulfurtransferase.
*envZ*	-1.5/-2.5/-2.1/-1.4	-envZ -ompR<	Sensory histidine kinase in two-component regulatory system with OmpR. Enzyme; Global regulatory functions
*ycbC*	-2.4/-2/-/-	-ycbC<	DUF218 superfamily protein.
*ftsN*	-1.4/-2.1/-/-	-rraA -menA -hslU -hslV -ftsN -cytR<	Essential cell division protein. Phenotype; Cell division

Non-significant log_2_FC values (q>0.0001 and/or log_2_CPM7<3) are indicated by a dash. For operon information >, < indicate direction of transcription; +*gene* indicates genes encoded on the forward strand; -*gene* indicates genes encoded on the reverse strand.

The cephalosporin β-lactam cefsulodin specifically inhibits PBP1a and PBP1b (Joseleau-Petit et al., 2007; Sarkar et al., 2012), so we tested for a synergistic relationship between cefsulodin and meropenem. The fractional inhibitory concentration index (FICI) is a measure of whether antibiotic combinations act synergistically (FICI<0.5), additively (FICI = 0.5 – 1), or do not interact (FICI>1) (Hall et al., 1983). The relative MICs for meropenem and cefsulodin were determined as 3.2 and 16 mg/L respectively. However, with cefsulodin at 8 mg/L, in one additive/synergy measurement the MIC for meropenem was 0.2 mg/L (FICI = 0.56) and in the other 0.1 mg/L (FICI = 0.53), indicating an additive effect. This result supports the identification of *lpoB* and *mrcB* as important for tolerance of meropenem and exemplifies the sensitivity of TraDIS-*Xpress* to predict relatively weak additive activity.

Knockout insertions in genes for both members of the FtsEX complex also increased susceptibility at all meropenem concentrations, as did insertions in *minC* and *minD* of the MinCDE complex. This experiment has highlighted the importance of cell division to meropenem tolerance, so targeting the machinery involved is a rational strategy for increasing the efficacy of β-lactams. Therefore, these two complexes are attractive targets that if inactivated will have detrimental effects at all downstream stages of septum formation and cell division.

This experiment has also highlighted the possibility of two-component signal transduction systems being important to β-lactam tolerance. Based on log_2_FC, the EnvZ/OmpR (osmotic stress response) and CpxRA (generalized membrane stress response) systems are the most promising targets. Intriguingly, these two systems are thought to be linked by the protein MzrA (Gerken et al., 2009). Another novel target that was revealed by the measurement of RNA interference effects is RNA gene expression, whether through directly inhibiting transcription of rRNA and tRNA genes or through inhibiting the stringent response by deactivating *relA*.

## Discussion

By employing TraDIS-*Xpress* to study the mechanisms involved in *E. coli* tolerance of meropenem, we have identified an extraordinary number of genes involved in susceptibility. Even by concentrating only on the most significant hits, we still identified 296 knockout insertions, 52 insertions with 5′ transcription-promoting activity and 48 insertions with 3′ transcription-inhibiting activity. This strongly suggests that the mechanism of action of meropenem reaches far beyond the canonical inhibition of peptidoglycan crosslinking by targeting penicillin binding proteins. It also indicates that there are multiple mechanisms by which resistance to meropenem could develop and raises the possibility that multiple subpopulations of cells arise upon meropenem exposure, in which different tolerance strategies are employed.

We identified four gene function categories of principal importance for meropenem tolerance. Unsurprisingly, cell envelope (particularly peptidoglycan) biogenesis was among these functions. However, the high degree of intersection with components of the divisome emphasizes the importance of cell division for meropenem efficacy. Furthermore, the fact that cell division genes showed highly significant log_2_FC at all meropenem concentrations indicates that peptidoglycan remodeling during cell division is the primary target of meropenem. Some transcription regulators were also important at all meropenem concentrations, reflecting a broad, generalized response to cell envelope perturbations caused by meropenem. However, ATP metabolism was only a significant function at higher meropenem concentrations.

A β-lactam-induced futile cycle of peptidoglycan synthesis and degradation has been proposed by Cho *et al.* (Cho et al., 2014). For the mecillinam-induced toxicity that they described, Slt (a lytic transglycosylase encoded by *slt*) was responsible for the turnover of nascent peptidoglycan. However, they did not identify the non-Slt protein(s) responsible for turnover upon cefsulodin treatment. In the present study, an alternative lytic transglycosylase, LdcA, and the amidase-activating EnvC were identified together with Slt. Mecillinam specifically targets PBP2, while meropenem has multiple targets (Kocaoglu and Carlson, 2015). Therefore, it is likely that a greater variety of uncrosslinked peptidoglycans are generated by meropenem and other β-lactams with multiple targets, necessitating the action of Slt, LdcA and one or both of AmiA and AmiB for their breakdown.

A recent report found that the mecillinam-induced peptidoglycan futile cycle leads to wide-scale downstream effects: futile cycling increases energy demand, causing an imbalance in central carbon metabolism, which generates reactive oxygen species and leads to disruption of the redox environment of the cell. This damages macromolecules such as DNA and proteins, leading to increased protein synthesis, which further increases energy demand. The eventual depletion of ATP and accumulated cell damage leads to death (Lobritz et al., 2022). Although so far only demonstrated for mecillinam, this runaway process of cell damage intriguingly implicates our other two principal gene functions of ATP metabolism and transcription regulation in a network of far-reaching downstream effects from β-lactam-induced cell envelope stress. Our results point to NADH:ubiquinone oxidoreductase and ATP synthase as the primary regulators of the metabolic response to peptidoglycan futile cycling. We observed that 5′ transcription-promoting insertions that increase expression of *nuo* genes increase survival, while for *atp* genes, knockout insertions are beneficial. A combination of these effects may be conducive to maintenance of the redox environment. However, our data point towards the importance of maintaining the membrane potential for meropenem tolerance; a consideration that was not directly addressed in (Lobritz et al., 2022).

Previous reports have also discussed the transcriptional response to β-lactams (Srivatsan and Wang, 2008; Battesti et al., 2011; Møller et al., 2016; Gray and Wenzel, 2020), including the roles played by the *cpxRA* (Guest et al., 2017) and *envZ/ompR* (Adler et al., 2016) two-factor systems. Clearly the intersecting relationships between β-lactam stress and all the mechanisms discussed here are extremely complex and will also be influenced by environmental and experimental conditions. For example, Guest *et al.* (Guest et al., 2017) found that CpxR inhibits *nuo* gene expression in response to envelope stress (although they did not test β-lactam antibiotic stress), while our results suggested that although the CpxRA system is involved in meropenem tolerance, increased expression can be beneficial under some conditions.

Recent work has demonstrated inter-linked effects on β-lactam tolerance of the membrane potential (Kaneti et al., 2016; Wang et al., 2021), ATP production (Shetty and Dick, 2018; Lindman and Dick, 2019), sugar metabolism (Thorsing et al., 2015), and the stringent response (Rodionov et al., 1995; Lai et al., 2017) in *E. coli* and other species. The membrane potential is also required for cell division (Strahl and Hamoen, 2010; Chimerel et al., 2012) and persister cell viability (Radzikowski et al., 2016; Manuse et al., 2021; Wang et al., 2021). We identified genetic components of all these processes as important contributors towards the response to meropenem stress. Often, we only observed significant selective pressures on these processes at or above the MIC, which provides compelling evidence that tolerant subpopulations of cells arose during the experiment.

Based on their significance at low meropenem concentrations, we identified proteins involved in peptidoglycan remodeling during cell division as potential targets for meropenem-synergizing drugs. We tested one such prediction, which revealed an additive effect of cefsulodin and meropenem due to inhibition of *mrcB*. Other previously reported inhibitors of proteins involved in cell division include bulgecin A, which inhibits *slt* (Templin et al., 1992) and a dithiazoline inhibitor of *ldcA* (Baum et al., 2005). As deletion of *slt* renders *E. coli* hypersensitive to mecillinam (Templin et al., 1992; Bendezú and De Boer, 2008), in agreement with the data presented here for meropenem, our data suggest a similar effect for knockouts of *ldcA, mepS* and amidases, which have similar functions.

Our data also suggested some new potential drug targets. For example, knockouts in FtsZ-associated divisome machinery such as *ftsEX* and *minCD* potentiate the effects of meropenem, even when peptidoglycan-recycling proteins are expressed at wild-type levels. We are not aware of specific inhibitors for these proteins, but they appear to be attractive targets for antibacterial drug discovery. The F_0_F_1_ ATP synthase and NADH:ubiquinone oxidoreductase might also be useful targets for drugs to modulate their expression or activity in such a way as to synergize with the effects of β-lactam antibiotics.

### Limitations of the study

The uniquely high-throughput approach of TraDIS-*Xpress* simultaneously assays upregulation, downregulation, and knockouts of every gene in the genome and is an extremely powerful way to identify multiple important mechanisms. However, the parallel nature of the assay does lead to convolution of results from different mechanisms and different populations of cells. This experiment provided evidence of tolerance arising through loss of the cell wall (*waa* and *wec* operons; *lpp*), modulation of metabolism and the membrane potential (*atp* and *nuo* operons) reduced growth rate (cell division machinery) and the stringent response (*spoT*/*relA*; tRNA genes). It has long been established that non-growing cells are intrinsically more resistant to β-lactams than when actively growing (Tuomanen, 1986; Lee et al., 2018), although growth reduction is not necessary for tolerance (Urbaniec et al., 2022). However, we cannot deduce how many separate subpopulations of tolerant cells emerged or which mechanisms gave rise to each. We also cannot determine the proportion of tolerant but (slowly) growing cells compared to persisters. Furthermore, TraDIS-*Xpress* measures a change in relative abundance of transposon insertion mutants. Therefore, it is challenging to distinguish between insertions that result in cells that can outgrow others in the population (an absolute increase in cell numbers) and those that simply “out-survive” others (e.g., by growing more slowly or becoming persisters: a relative increase in cell numbers).

## Conclusions

We have used TraDIS-*Xpress* to identify many genes that are important for *E. coli* survival of meropenem stress and found that four principal gene functions of cell envelope biogenesis, cell division, ATP metabolism, and transcription regulation encompass the most significant and important responses. Some identified genes are involved in well-established mechanisms, validating the use of TraDIS-*Xpress*, while others are involved in downstream damage-limitation responses and support recent advances in our understanding of the systemic effects of β-lactams. We were also able to add to the list of genes known to be involved in meropenem susceptibility and tolerance, and to predict drug targets to potentiate the effects of meropenem. Similar experiments using different β-lactam antibiotics will enable comparisons to be made, to better understand the mode of action of each drug and to identify common and/or unique targets between drugs. Our data strongly suggest that multiple routes to meropenem tolerance are important. Therefore, identification and further characterization of the individual pathways leading to meropenem tolerance would be illuminating.

## Data availability statement

The nucleotide sequencing data for this study can be found in the Array Express repository under accession number E-MTAB-11802.

## Author contributions

NT performed data analysis, produced figures, and wrote the manuscript; AT and MY designed and performed the experiments and curated the data; SB and ML helped to curate the data and performed computational analyses; MW and IC helped design experiments and provided managerial oversight. All authors contributed to the article and approved the submitted version.

## Funding

This work was funded by the Biotechnology and Biological Sciences Research Council Institute Strategic Programme Microbes in the Food Chain BB/R012504/1 and its constituent project BBS/E/F/000PR10349.

## Conflict of interest

The authors declare that the research was conducted in the absence of any commercial or financial relationships that could be construed as a potential conflict of interest.

## Publisher’s note

All claims expressed in this article are solely those of the authors and do not necessarily represent those of their affiliated organizations, or those of the publisher, the editors and the reviewers. Any product that may be evaluated in this article, or claim that may be made by its manufacturer, is not guaranteed or endorsed by the publisher.
