# Supplementary material for: A whole-genome assay identifies four principal gene functions that confer tolerance of meropenem stress upon Escherichia coli
Source: Front Antibiot. 2022 Sep 16;1:957942. doi: 10.3389/frabi.2022.957942 (PMC11731830; doi:10.3389/frabi.2022.957942)
Supplement: Supplementary file 2 [file Table_2.docx]

**Supplementary Table 2:** Gene Functional Classification groups based on all knockout insertions with q < 0.0001 and log2CPM > 3. Enrichment score is the geometric mean of the p-values for all group members, represented in -log scale (higher values indicate consistently more significant enrichment within the group vs MG1655 whole genome). Gene names in bold type are members of more than one group.

| Group # | N^o.^ of genes | Enrichment score | Group members | Function summary |
| --- | --- | --- | --- | --- |
| 1 | 7 | 3.27 | *atpA****B****CD****E****GH* | ATP synthase |
| 2 | 5 | 2.65 | *waaBCFGQ* | Lipopolysaccharide core region biosynthesis |
| 3 | 10 | 2.11 | *acrA*  *cpxR*  *fis*  ***gadE***  ***gcvA***  ***nagC***  *ompR*  ***phoP***  *rcsB*  ***zur*** | Transcription regulation/two-component systems |
| 4 | 13 | 2.06 | *arcB*  *barA*  ***citA***  ***cyoE***  ***dgkA***  *envZ*  *glnL*  *phoQ*  *rcsC,****D***  ***ybcI***  ***ygiZ***  *zraS* | Signal transduction/two-component systems |
| 5 | 6 | 1.72 | *nuoCFG****KLN*** | Respiratory complex I (NADH:ubiquinone oxidoreductase) |
| 6 | 22 | 1.68 | *chpS*  *cra*  *cytR*  *deoR*  *frlR*  *fur*  ***gadE***  *gadX*  ***gcvA***  *hdfR*  *higA*  *mlc*  *mqsA*  *mraZ*  *mtlR*  ***nagC***  ***phoP***  *rbsR*  *slyA*  *yebK*  *yen*  ***zur*** | Transcription regulation |
| 7 | 8 | 1.46 | *acrA*  *bamB,E*  *lpp*  *mdtE*  *mepS*  *ygdI*  *ysaB* | Lipoproteins involved in outer membrane and murein sacculus biogenesis |
| 8 | 6 | 1.04 | *cydC,D*  *ftsE*  *mlaF*  *pstB*  *sapD* | ATP binding proteins involved in metabolism and cell division |
| 9 | 45 | 0.80 | *acrB*  *ampG*  ***atpB,E***  ***citA***  *corA*  *csrD*  *cydA,B*  *cyoBC****E***  *dedD*  ***dgkA***  *feoB*  *ftsLNWX*  *gtrS*  *lpxT*  *malG*  *metI*  *mlaE*  *nhaA*  ***nuoKLN***  *opgH*  *panF*  *pitA*  *pstA,C*  ***rcsD***  *sapC*  *tatC*  *trkH*  *wecA*  *wzxE*  *xanP*  ***ybcI***  ***ygiZ***  *yhfK*  *ykgH*  *yrbG* | Transmembrane/cell membrane/ATP synthesis/cell cycle/oxidative phosphorylation |
